# [*N*,*N*-Bis(2-hy­droxy­eth­yl)di­thio­carbamato-κ^2^ *S*,*S*′]bis­(tri­phenyl­phosphane-κ*P*)copper(I) chloro­form monosolvate: crystal structure, Hirshfeld surface analysis and solution NMR measurements

**DOI:** 10.1107/S2056989016017837

**Published:** 2016-11-15

**Authors:** Sang Loon Tan, Chien Ing Yeo, Peter J. Heard, Geoffrey R. Akien, Nathan R. Halcovitch, Edward R. T. Tiekink

**Affiliations:** a Research Centre for Crystalline Materials, Faculty of Science and Technology, Sunway University, 47500 Bandar Sunway, Selangor Darul Ehsan, Malaysia; b Office of the Provost, Sunway University, 47500 Bandar Sunway, Selangor Darul Ehsan, Malaysia; c Department of Chemistry, Lancaster University, Lancaster LA1 4YB, UK

**Keywords:** crystal structure, copper, di­thio­carbamate, hydrogen bonding, Hirshfeld surface analysis, NMR

## Abstract

The title compound, [Cu(C_5_H_5_NO_2_S_2_)(C_18_H_15_P)_2_]·CHCl_3_, features a tetra­hedrally coordinated Cu^I^ atom within a P_2_S_2_ donor set defined by two phosphane P atoms and by two S atoms derived from a symmetrically coordinating di­thio­carbamate ligand. Both intra- and inter­molecular hy­droxy-O—H⋯O(hydroxy) hydrogen bonding is observed: the former closes an eight-membered {⋯HOC_2_NC_2_O} ring, whereas the latter connects centrosymmetrically related mol­ecules into dimeric aggregates *via* eight-membered {⋯H—O⋯H—O}_2_ synthons. The complex mol­ecules are arranged to form channels along the *c* axis in which reside the chloro­form mol­ecules, being connected by Cl⋯π(arene) and short S⋯Cl [3.3488 (9) Å] inter­actions. The inter­molecular inter­actions have been investigated further by Hirshfeld surface analysis, which shows the conventional hydrogen bonding to be very localized with the main contributors to the surface, at nearly 60%, being H⋯H contacts. Solution NMR studies indicate that whilst the same basic mol­ecular structure is retained in solution, the tri­phenyl­phosphane ligands are highly labile, exchanging rapidly with free Ph_3_P at room temperature.

## Abstract 2

A tetra­hedral CuP_2_S_2_ coordination geometry is found for the Cu^I^ ion in the title compound. The di­thio­cabamate ligand forms symmetric Cu—S bonds. In the crystal, supra­molecular dimers of complex mol­ecules are connected *via* eight-membered {⋯H—O⋯H—O}_2_ synthons. In addition, the chloro­form mol­ecule participates in Cl⋯π(arene) and S⋯Cl inter­actions.

## Chemical context   

The motivation to prepare bis­(phosphane)copper(I) di­thio­carbamates of general formula (*R*_3_P)_2_Cu(S_2_CN*R*′*R*′′) (*R*, *R*′, *R*′′ = alkyl, ar­yl) largely stems from the versatile biological properties exhibited by these types of compounds (Skrott & Cvek, 2012 ▸; Biersack *et al.*, 2012 ▸) and metal di­thio­carbamates in general, as summarized in a recent review (Hogarth, 2012 ▸). At present, research continues to develop promising anti-microbial agents in light of the growing prevalence of bacterial infections and threats associated with drug-resistant bacteria (Verma & Singh, 2015 ▸; Onwudiwe *et al.*, 2016 ▸). In our recent efforts to develop anti-microbial agents, phosphanegold(I) di­thio­carbamates, *R*_3_PAu[S_2_CN(*i*Pr)CH_2_CH_2_OH], were prepared and these compounds demonstrated prominent and distinctive anti-microbial activity against a broad range of Gram-positive and Gram-negative bacteria, dependent on the type of P-bound substituent employed (Sim *et al.*, 2014 ▸). A distinct structure–activity relationship was noted in that when *R* = Et, the compound was potent against a broad range of Gram-positive and Gram-negative bacteria, whereas the *R* = Ph and Cy compounds showed specific activity against Gram-positive bacteria. Even greater, broad-range activity is apparent in tri­ethyl­phosphanegold(I) di­alkyl­dithio­carbamates (Chen *et al.*, 2016 ▸). The above prompted an exploration of the anti-bacterial activity of related copper(I) and silver(I) deriv­atives, as these metals are known to possess noteworthy potential as anti-microbial agents (Losasso *et al.*, 2014 ▸). Thus, a series of phosphanecopper(I) and silver(I) compounds of general formula (Ph_3_P)_2_ *M*[S_2_CN(*R*)CH_2_CH_2_OH] for *M* = Cu and Ag, and *R* = Me, *i*Pr and CH_2_CH_2_OH, were prepared and evaluated for their anti-microbial activities (Jamaludin *et al.*, 2016 ▸). While none of the studied compounds exhibited activity against Gram-negative bacteria, they were found to be selectively potent against Gram-positive bacteria. Following new syntheses to evaluate further the potential of this class of compounds, crystals became available for the title complex, (Ph_3_P)_2_Cu[S_2_CN(CH_2_CH_2_OH)_2_] (I), as its 1:1 chloro­form solvate. Herein, the crystal and mol­ecular structures of (I)·CHCl_3_ are described along with an analysis of its Hirshfeld surface. Finally, some non-standard, *e.g*. variable temperature, NMR measurements are presented in order to gain insight into the solution structure.
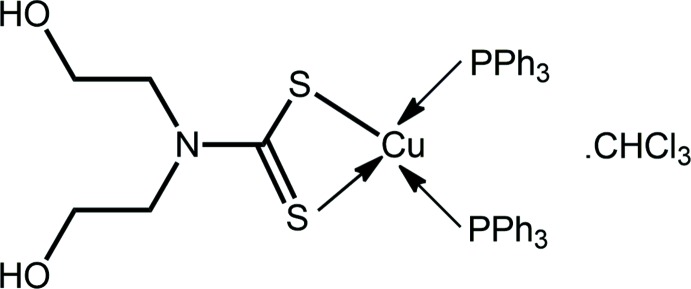


## Structural commentary   

**Figure 1:**
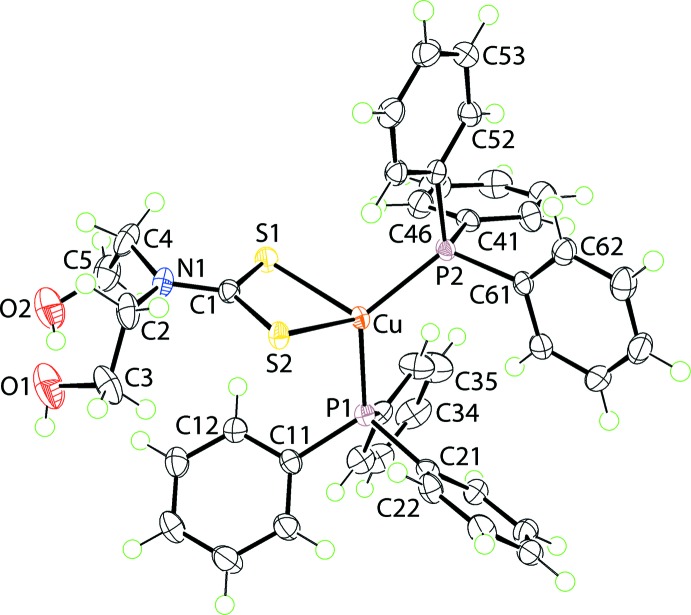
The mol­ecular structure of the complex in (I)·CHCl_3_, showing the atom-labelling scheme and displacement ellipsoids at the 70% probability level. The solvent CHCl_3_ mol­ecule is omitted.

The mol­ecular structure of the complex in (I)·CHCl_3_ is shown in Fig. 1 ▸ and selected geometric parameters are collected in Table 1 ▸. The copper atom is bound by two di­thio­carbamate-S atoms and two phosphane-P atoms. The di­thio­carbamate ligand is coordinating in a symmetric mode with Δ(Cu—S) = 0.042 Å, being the difference between the Cu—S_long_ and Cu—S_short_ bond lengths. This near equivalence in Cu—S bond lengths is reflected in the experimental equivalence of the associated C1—S1, S2 bond lengths. A small disparity, *i.e*. 0.02 Å, is noted in the Cu—P bond lengths. The resulting P_2_S_2_ donor set defines an approximate tetra­hedral geometry. A measure of tetra­hedral *vs* square-planar geometry is the value of τ_4_ (Yang *et al.*, 2007 ▸) with values of 1.0 and 0.0° corres­ponding to ideal tetra­hedral and square planar geometries, respectively. In the case of the complex in (I)·CHCl_3_, the value computes to 0.80. Distortions from the ideal tetra­hedral geometry are clearly related to the acute angle subtended by the di­thio­carbamate ligand and the wide angle subtended by the bulky tri­phenyl­phosphane ligands, Table 1 ▸.

**Figure 2:**
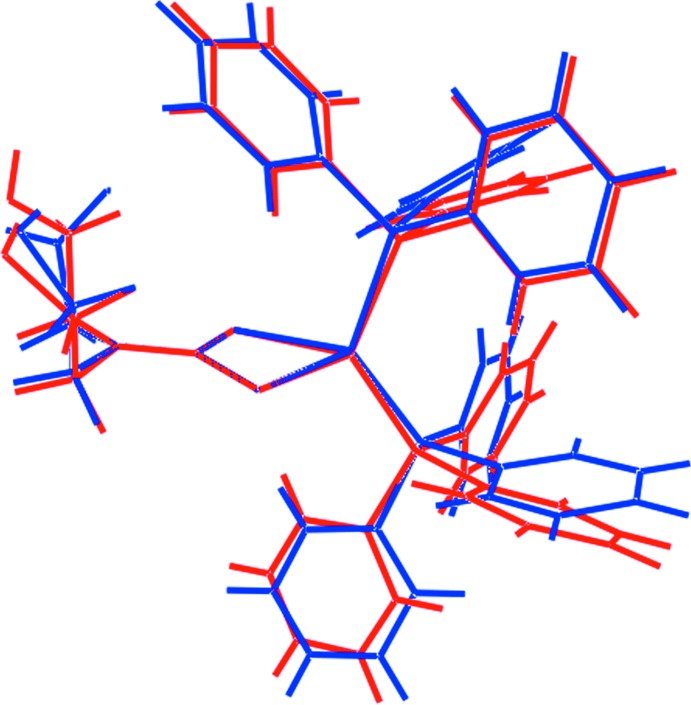
Overlay diagram of (I)·CHCl_3_ (red image) and (I)·Ph_3_P (blue). The mol­ecules have been overlapped so the chelate rings are coincident. The CHCl_3_ and Ph_3_P mol­ecules have been omitted.

The structure of (I) has also been determined in its 1:1 co-crystal with PPh_3_ (Jian *et al.*, 2000 ▸), hereafter (I)·Ph_3_P, and key geometric parameters for this structure are also included in Table 1 ▸. Inter­estingly, within pairs of comparable bond lengths, those in (I)·PPh_3_ are systematically longer. However, the value of Δ(Cu—S) is slightly less at 0.034 Å. The value of τ_4_ is identical at 0.80. An overlay diagram for (I) in each of (I)·CHCl_3_ and (I)·PPh_3_ is shown in Fig. 2 ▸ which confirms the very similar conformations adopted for (I) in both structures.

## Supra­molecular features   

**Figure 3:**
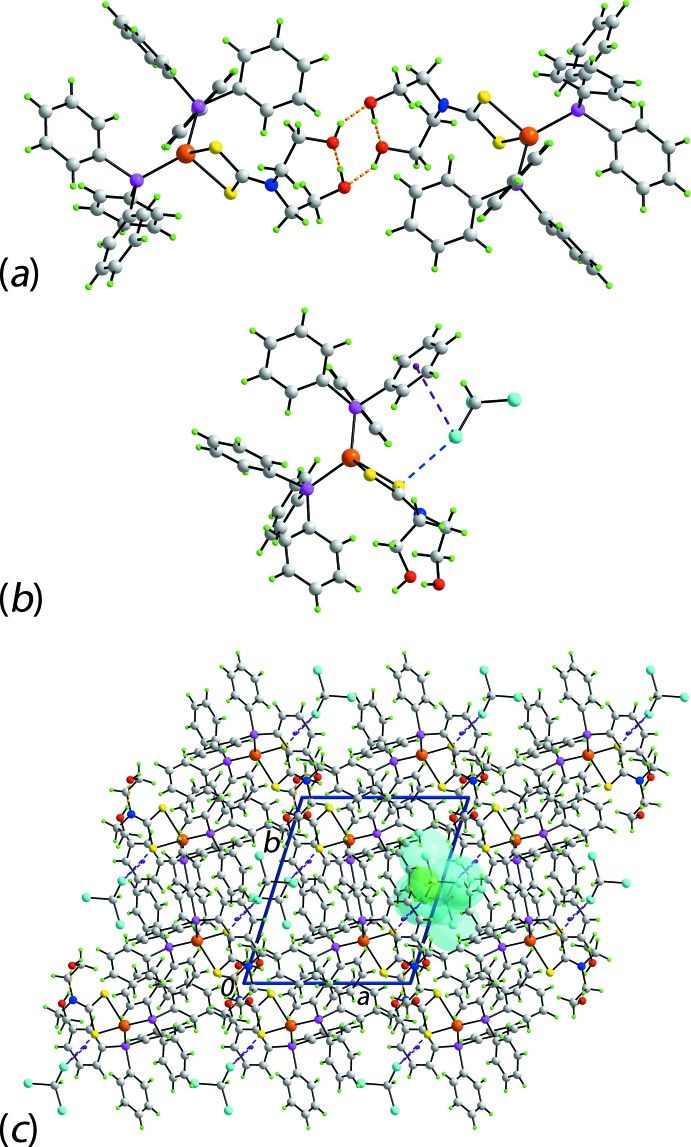
Mol­ecular packing in (I)·CHCl_3_: (*a*) supra­molecular dimer sustained by hy­droxy-O—H⋯O(hy­droxy) hydrogen bonding shown as orange dashed lines, (*b*) a view of the inter­actions between the complex and solvent mol­ecules with the Cl⋯π(arene) and Cl⋯S inter­actions shown as purple and blue dashed lines, respectively, and (*c*) a view of the unit-cell contents in projection down the *c* axis, with chloro­form mol­ecules occupying one channel highlighted in space-filling mode.

Geometric parameters describing the salient inter­molecular inter­actions in the crystal of (I)·CHCl_3_ are collated in Table 2 ▸. There are two types of hy­droxy-O—H⋯O(hy­droxy) hydrogen bonding in the mol­ecular packing, one intra­molecular and the other inter­molecular. The former has hy­droxy-O2—H as the donor and the hy­droxy-O1 as the acceptor, and closes an eight-membered {⋯HOC_2_NC_2_O} ring. The key feature of the mol­ecular packing is the presence of hy­droxy-O—H⋯O(hy­droxy) hydrogen bonding which connects centrosymmetriclly-related mol­ecules into dimeric aggregates *via* eight-membered {⋯H—O⋯H—O}_2_ synthons, encompassing the intra­molecular hy­droxy-O—H⋯O(hydroxy) hydrogen bonds, Fig. 3 ▸ *a*. The only other identifiable directional inter­actions within standard distance criteria (Spek, 2009 ▸) involve the chloro­form mol­ecule. Thus, a chloro­form-Cl3⋯π(arene) inter­action is noted, Table 2 ▸. In addition, there is evidence for a close S1⋯Cl3 contact, *i.e*. involving the same chlorine atom as in the just mentioned Cl⋯π(arene) inter­action. The separation of 3.3488 (9) Å is about 0.2 Å less than the sum of their van der Waals radii (Spek, 2009 ▸). In a very recent and exhaustive review of halogen bonding (Cavallo *et al.*, 2016 ▸), it was mentioned that sulfur is well known to function as an acceptor in *R*—*X*⋯S synthons. The inter­actions involving the chloro­form mol­ecule are highlighted in Fig. 3 ▸ *b*. Globally, mol­ecules of the copper(I) complex pack to define channels parallel to the *c* axis in which reside the solvent mol­ecules, Fig. 3 ▸ *c*. Given the presence of Ph_3_P ligands in (I)·CHCl_3_, evidence was sought for phenyl-embraces (Dance & Scudder, 1995 ▸). While none was apparent for the P1-phosphane, centrosymmetrically related P2-phosphane ligands approach each other in this manner to generate a sixfold phenyl-embrace. The closest inter­actions between the phosphane residues in this embrace is a pair of edge-to-face-phen­yl—H⋯π(arene) inter­actions, *i.e*. C63—H63⋯π(C51–C56)^i^ = 3.25 Å with an angle at H62 of 133°; symmetry operation (i): 1 − *x*, 1 − *y*, 1 − *z*.

## Hirshfeld surface analysis   

**Figure 4:**
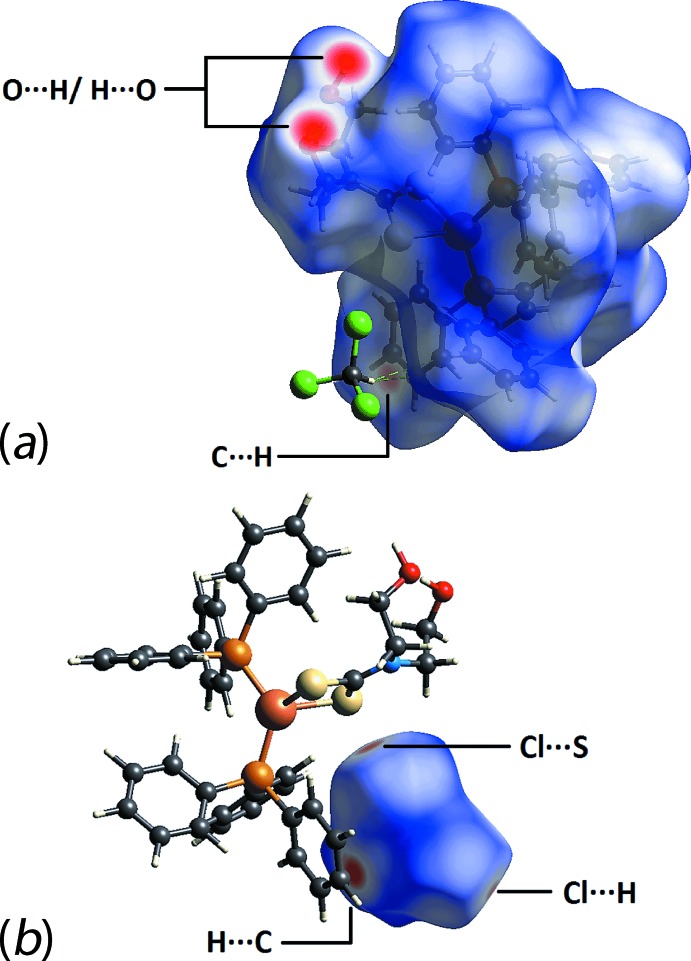
Comparison of the Hirshfeld surfaces of (*a*) mol­ecule (I) in (I)·CHCl_3_ and (*b*) CHCl_3_ in (I)·CHCl_3_, highlighting inter­molecular inter­actions formed with the other component of the structure. The Hirshfeld surfaces were mapped over *d*_norm_ within the range −0.572 to 1.457 Å.

The protocols for the Hirshfeld surface analysis were as described recently (Yeo *et al.*, 2016 ▸). In the present study, analyses were conducted on the following three species: (I) in (I)·CHCl_3_, (I)·CHCl_3_ and CHCl_3_ alone. Hirshfeld surface analysis provides visualization on the existence of any inter­molecular inter­actions within close proximity in a crystal structure, for which contact distances shorter than the sum of the respective van der Waals radii appear red while at distances equal or longer than this would be white and blue in appearance, respectively. Figs. 4 ▸ *a* and *b* show Hirshfeld surfaces mapped over *d*_norm_ for (I) and CHCl_3_, respectively. The former image exhibits intense red spots on the surface near the hy­droxy­ethyl substituents which are correlated with the strong O—H⋯O hydrogen bonding. Apart from these dominant inter­actions, several other red spots attributed to the close contacts between the complex and chloro­form mol­ecules, *i.e*. C⋯H/H⋯C, S⋯Cl/Cl⋯S and H⋯Cl/Cl⋯H, are evident in Fig. 4 ▸ *a* and *b*.

**Figure 5:**
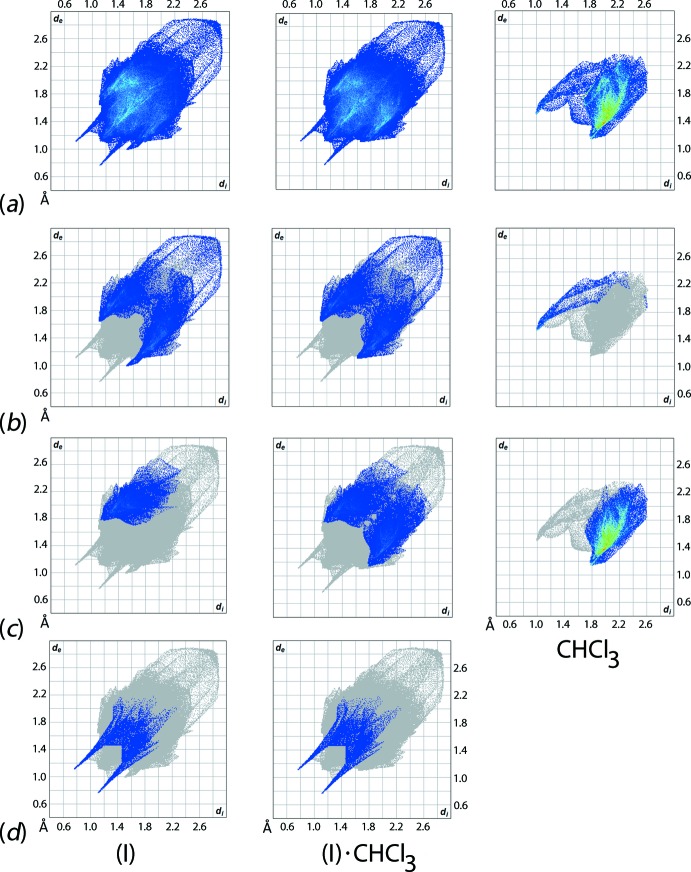
Comparison between (I) in (I)·CHCl_3_, (I)·CHCl_3_ and CHCl_3_ of the (*a*) full two-dimensional fingerprint plots, and the plots delineated into (*b*) C⋯H/H⋯C, (*c*) Cl⋯H/H⋯Cl and (*d*) O⋯H/H⋯O contacts.

The combination of *d*_i_ and *d*_e_ distances resulted in two-dimensional cuttlefish- and chicken wing-like fingerprint plots for (I), (I)·CHCl_3_ and CHCl_3_, Fig. 5 ▸ *a*, which may be decomposed into several essential close contacts as shown in Fig. 5 ▸ *b*–*d*. In general, complex (I) and its chloro­form solvate exhibit almost identical profiles except that the pincer form of (I) in its decomposed fingerprint plot delineated into C⋯H/H⋯C contacts shows two different tips at *d*_e_ + *d*_i_ ∼ 2.5 Å and ∼2.7  Å in contrast to the pincer form of (I)·CHCl_3_ with a pair of symmetrical tips at *d*_e_ + *d*_i_ ∼ 2.7 Å when the solvate is considered as a single entity. The close contact distance (*d*_e_ + *d*_i_ ∼ 2.5 Å), which is shorter than the sum of van der Waals radii of 2.9 Å (Spek, 2009 ▸), is also reflected in the lancet blade-like fingerprint plot of the solvent mol­ecule corres­ponding to the Cl—H⋯C(π) inter­action. The H⋯Cl/Cl⋯H contact, on the other hand, contributes to the half-pincer form in the decomposed fingerprint plot of (I) and develops into the full pincer form in (I)·CHCl_3_, both with *d*_e_ + *d*_i_ ∼ 2.9 Å that is very close to the sum of van der Waals radii (2.95 Å). As expected, O⋯H/H⋯O contacts constitute the strongest among all inter­actions with *d*_e_ + *d*_i_ ∼ 1.9 Å (*cf*. the sum of van der Waals radii of 2.75 Å) in the forceps form of both decomposed fingerprint plots of (I) and (I)·CHCl_3_, Fig. 5 ▸ *d*. Based on the asymmetric fingerprint patterns of the C⋯H/H⋯C and Cl⋯H/H⋯Cl contacts, Fig. 5 ▸ *b* and *c*, and the symmetric pattern of the O⋯H/H⋯O contacts, Fig. 5 ▸ *d*, it may be concluded that two complex mol­ecules are very closely associated, as shown in Fig. 3 ▸ *a*, and these are flanked by two CHCl_3_ mol­ecules, highlighted in Fig. 3 ▸ *b*.

**Figure 6:**
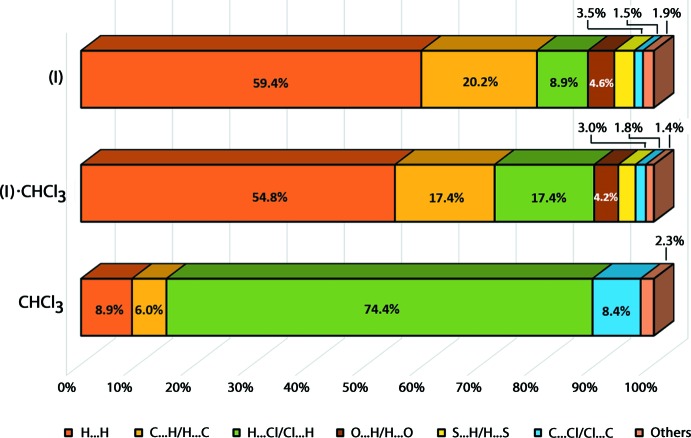
Percentage contributions of the different close contacts to the Hirshfeld surface of (I) in (I)·CHCl_3_, (I)·CHCl_3_ and CHCl_3_.

The qu­anti­fication on the distribution of each of the contacts to the Hirshfeld surface reveals that H⋯H, C⋯H/H⋯C and H⋯Cl/Cl⋯H are the three main components for (I) in (I)·CHCl_3_, with the corresponding contributions of *ca* 59.4, 20.2 and 8.9%, respectively, Fig. 6 ▸. Despite this, not all of these contacts result in meaningful inter­actions based on the comparison between *d*_e_ + *d*_i_ contact distances and the sum of the van der Waals radii. This sequence is followed by O⋯H/ H⋯O contacts which form the fourth most dominant inter­actions with a contribution of approximately 4.6% to the overall Hirshfeld surface. In general, there is not much deviation of the topological distribution between (I) and (I)·CHCl_3_ except that the contribution from H⋯Cl/Cl⋯H increases by nearly twofold upon the inclusion of the solvent mol­ecule in (I)·CHCl_3_. As for the chloro­form mol­ecule, H⋯Cl/Cl⋯H makes the major contribution at 74.4%, followed by 8.9% from H⋯H and 8.4% from H⋯Cl/Cl⋯H; the remaining contributions from other minor contacts.

As mentioned previously, Cl⋯*π*(arene) and S⋯Cl inter­actions are formed by the chloro­form mol­ecule. In order to gain insight into the charge distribution and rationalize these close contacts, the electrostatic potential (ESP) was mapped over the Hirshfeld surface by *ab initio* Hartree–Fock (HF) quantum modelling with the 6-31G(d) basis set, as this represents the best possible level of theory and basis set functions in this study so as to keep the accuracy and computational cost at manageable level.

**Figure 7:**
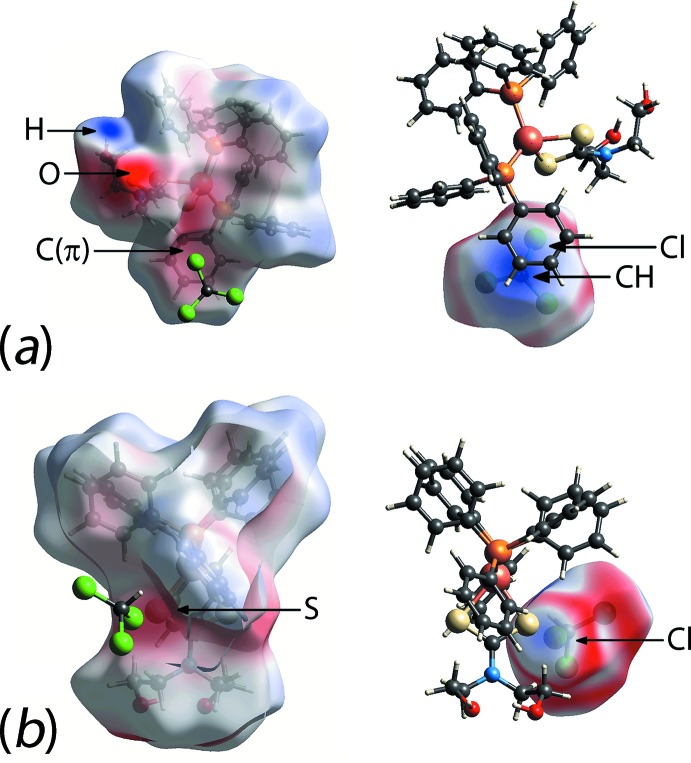
Electrostatic potential (ESP) mapped over the Hirshfeld surfaces of the complex mol­ecule (I) (left) and CHCl_3_ (right), showing the attraction between the electronegative (red) and electropositive (blue) sites for (*a*) Cl⋯π(arene) and (*b*) S⋯Cl inter­actions, respectively. The ESP was mapped onto the Hirshfeld surface within the isocharge value of −0.119 to 0.164 a.u. by the *ab initio* Hartree–Fock (HF) quantum modelling approach with the 6-31G(d) basis set.

As shown in Fig. 7 ▸ *a*, a phenyl ring of the complex mol­ecule exhibits mild electronegative character as evidenced from the pale-red spot on the ESP map in contrast to the strong electropositive character about CHCl_3_, being intense-blue. The electropositive character of the methine group extends slightly beyond the chloro atom approaching its equatorial ring of the negative charge region, hence establishing the weak Cl⋯*π*(arene) inter­action with *d*_e_ + *d*_i_ ∼ 3.3 Å being slightly less than the sum of van der Waals radii of 3.45 Å. The S⋯Cl halogen bond, on the other hand, is established through the highly directional inter­action between the electronegative sulfur of (I) and the *σ*-hole of the chloro atom of CHCl_3_ with weak electropositive character, Fig. 7 ▸ *b*. The electropositive character of the *σ*-hole results from the electron deficiency in the outer lobe of the *p* orbital (non-bonded) when a half-filled *p* orbital of a halogen participates in the formation of covalent bond (Clark *et al.*, 2007 ▸).

## NMR Study   

FT NMR spectra were recorded on a Bruker AVANCE III 400 MHz spectrometer, operating at 400.13, 100.61 and 161.98 MHz, respectively, for ^1^H, ^13^C and ^31^P. Spectra were indirectly referenced to the solvent deuterium lock shift; chemical shifts are quoted relative to TMS and 85% H_3_PO_4_. Probe temperatures were controlled by a standard variable temperature unit and are considered accurate to within ±1 K. Spectra were acquired on approximately 14 mmol solutions of (I) in each of CD_2_Cl_2_, *d*_6_-DMSO and CDCl_2_CDCl_2_.

The ambient temperature (298 K) ^1^H NMR spectra of (I) display the expected signals due to the tri­phenyl­phosphine and di­thio­carbamate ligands. The spectra are qualitatively identical in all three solvents, with the only significant differences being the position of the –OH signal of the di­thio­carbamate ligand.

The aromatic region of the ^1^H spectrum in *d*_6_-DMSO shows two multiplets at *ca* 7.39 ppm (6 H) and 7.28 ppm (24 H) attributable to Ph-H atoms of the tri­phenyl­phosphine ligands. A sharp singlet observed at 8.32 ppm (1 H) was assigned to CHCl_3_, as seen in the X-ray crystal structure analysis. The di­thio­carbamate moiety displays a single set of resonances, indicating the two –CH_2_CH_2_OH groups are chemically equivalent. The –OH groups display a triplet at 4.80 ppm (^3^ *J*_HH_ = 5.3 Hz), which disappears on the addition of D_2_O. The methyl­ene hydrogen atoms display a triplet at 3.96 ppm (^3^ *J*_HH_ = 6.4 Hz) and a pseudo quartet at 3.65 ppm, assignable to NCH_2_– and –CH_2_OH, respectively. On the addition of D_2_O, the quartet collapses to a triplet.

The ^13^C{^1^H} spectra in each of the solvents are also qualitatively identical. In *d*_6_-DMSO solution, the carbon atoms of the tri­phenyl­phospine ligands give rise to four resonances at 134.6 ppm (very weak, *d*, ^1^ *J*_PC_ ∼22 Hz, C_*ipso*_), 133.6 ppm (*d*, ^2^ *J*_PC_ = 12 Hz, C_*ortho*_), 130.1 ppm (*s*, C_*para*_) and 128.9 ppm (*d*, ^3^ *J*_PC_ = 5.70 Hz, C_*meta*_). The di­thio­carbamate ligand shows two signals due to the methyl­ene carbon atoms at 58.7 ppm (NCH_2_—) and 56.0 ppm (—CH_2_OH), respectively. The quaternary carbon atom of the di­thio­carbamate was not observed.

The ambient temperature ^31^P{^1^H} spectrum in CD_2_Cl_2_ displays as single, broad resonance at −1.55 ppm (Δv_1/2_ = 280 Hz). The line broadening is attributed to rapid relaxation of Cu *via* the quadrupole relaxation (QR) mechanism. Quadrupole relaxation is strongly temperature dependent: the rate of relaxation increases as the temperature decreases. On cooling, the signal sharpens progressively: Δv_1/2_ (203 K) ∼35 Hz. The sharpening presumably arises because of the effective ‘decoupling’ of the ^65^Cu–^31^P and ^63^Cu–^31^P scalar couplings as the rate of (Cu) relaxation increases (Grace *et al.*, 1970 ▸). The addition of *ca* 2 mg (0.9 equivalents) of tri­phenyl­phosphine at ambient temperature, to putatively give (I)·PPh_3_, gives a single, broad peak at *ca* −3 ppm, which is between the chemical shifts of pure (I) and free PPh_3_ (*ca* −6 ppm), indicating rapid exchange of the tri­phenyl­phosphine ligands.

In an attempt to resolve the Cu-P *J* couplings, ^31^P{^1^H} spectra were recorded in CDCl_2_CDCl_2_ solution at elevated temperatures (to reduce the rate of QR). However, no significant changes were observed in the line widths on elevating the temperature to 328 K, and any Cu–P coupling, if not lost through reversible ligand dissociation, remained unresolved. There was no evidence of decomposition at higher temperatures in this solvent.

There are two key conclusions from the foregoing. Firstly, the experiments with D_2_O proving exchange of the hy­droxy-H atom indicates that this atom is labile, suggesting functionalization at this group should, in principle, be feasible. Secondly, the presence of additional Ph_3_P in solution does not result in displacement of the di­thio­carbamate ligand nor force a monodentate mode of coordination proving the stability of complex (I) in each of (I)·CHCl_3_ and (I)·PPh_3_, and in solution.

## Database survey   

The structural chemistry of (*R*_3_P)_2_Cu(S_2_CN*R*′*R*′′) compounds was summarized very recently (Jamaludin *et al.*, 2016 ▸). In all, there are eight examples now available in the literature, namely {(Ph_3_P)_2_Cu[S_2_CN(Me)(CH_2_CH_2_OH)]}·CH_2_Cl_2_ Jamaludin *et al.*, 2016 ▸), [(Ph_3_P)_2_Cu{S_2_CN(CH_2_CH_2_OH)_2_}]·PPh_3_ (Jian *et al.*, 2000 ▸), [(Ph_3_P)_2_Cu{S_2_CN(*n*-Pr)_2_}]·CH_2_Cl_2_ (Xu *et al.*, 2001 ▸), [(Ph_3_P)_2_Cu{S_2_CN(CH_2_CH_2_)_2_S}]·CH_2_Cl_2_ (Gupta *et al.*, 2013 ▸), [(Ph_3_P)_2_Cu{S_2_CN(CH_2_CH_2_)_2_NPh}] (Gupta *et al.*, 2013 ▸), [(Ph_3_P)_2_Cu{S_2_CN(Me)CH_2_Ph}]·CH_2_Cl_2_ (Kumar *et al.*, 2009 ▸) and [(Ph_3_P)_2_Cu{S_2_CN(CH_2_Ph)(CH_2_py-4)}]·2H_2_O (Rajput *et al.*, 2012 ▸). Inter­estingly, all but one structure co-crystallizes with another mol­ecule, solvent or otherwise, perhaps indicating inefficient mol­ecular packing for these mol­ecules. The P_2_S_2_ donor sets all eight compounds approximate tetra­hedral angles with the range of τ_4_ values being a low 0.78 in {(Ph_3_P)_2_Cu[S_2_CN(Me)(CH_2_CH_2_OH)]}·CH_2_Cl_2_ (Jamaludin *et al.*, 2016 ▸) to a high of 0.85 in [(Ph_3_P)_2_Cu{S_2_CN(CH_2_CH_2_)_2_S}]·CH_2_Cl_2_ (Gupta *et al.*, 2013 ▸), the narrow range emphasizing the similarity in the mol­ecular structures/geometries.

## Synthesis and crystallization   

All chemicals and solvents were used as purchased without purification, and all reactions were carried out under ambient conditions. The melting point was determined on a Biobase automatic melting point apparatus MP450. The IR spectrum was obtained on a Perkin Elmer Spectrum 400 FT Mid-IR/Far-IR spectrophotometer from 4000 to 400 cm^−1^; abbreviations: *br*, broad; *m*, medium; *s*, strong.

Preparation of (I)·CHCl_3_: tri­phenyl­phosphine (Alfa Aesar, 2 mmol, 0.524 g) in aceto­nitrile (Merck, 10 ml) was added to copper(I) chloride (Sigma Aldrich, 1 mmol, 0.099 g) in aceto­nitrile (10 ml), followed by addition of a dispersion of potassium bis­(2-hy­droxy­eth­yl)di­thio­carbamate (1 mmol, 0.219 g) in aceto­nitrile (15 ml), prepared from the standard procedures (Jamaludin *et al.*, 2016 ▸). The resulting mixture was stirred for 2 h at room temperature. Chloro­form (Merck, 35 ml) was added to the reaction mixture and it was left for slow evaporation at room temperature. Yellow blocks of (I)·CHCl_3_ were obtained after one day. Yield: 0.699 g (91%). M.p. 423.8–424.5 K. IR (cm^−1^): 3268 (*br*) (OH), 1433 (*s*) (C—N), 1168 (*m*), 990 (*s*) (C—S).

## Refinement   

Crystal data, data collection and structure refinement details are summarized in Table 3 ▸. Carbon-bound H atoms were placed in calculated positions (C—H = 0.95–1.00 Å) and were included in the refinement in the riding-model approximation, with *U*_iso_(H) set to 1.2*U*_eq_(C). Refinement of the O-bound H atoms proved unstable so these atoms were fixed in the model in the positions revealed by a difference Fourier map, with *U*_iso_(H) = 1.5*U*_eq_(O). The maximum and minimum residual electron density peaks of 1.97 and 1.93 e Å^−3^, respectively, were located 0.78 and 0.62 Å from the Cl1 atom. While this feature of the difference map might indicate disorder, additional peaks that might be anti­cipated for the other atoms in the disordered component of chloro­form mol­ecule were not evident. This, plus the observation that the anisotropic displacement parameters of the atoms comprising the chloro­form mol­ecule exhibited no unusual features, suggest the residual electron densities have limited chemical significance. Finally, owing to poor agreement, four reflections, *i.e*. (326), (1
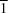
5), (666) and (1
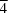
2) , were omitted from the final cycles of refinement.

## Supplementary Material

Crystal structure: contains datablock(s) I, global. DOI: 10.1107/S2056989016017837/hb7632sup1.cif

Structure factors: contains datablock(s) I. DOI: 10.1107/S2056989016017837/hb7632Isup2.hkl

CCDC reference: 1515483

Additional supporting information: crystallographic information; 3D view; checkCIF report

## Figures and Tables

**Table 1:** Geometric data (Å, °) for (I) in (I)·CHCl_3_ and (I) in its 1:1 Ph_3_P co-crystal

Parameter	(I) in (I)·CHCl_3_	(I) in (I)·PPh_3_^*a*^
Cu—S1	2.3791 (6)	2.3948 (12)
Cu—S2	2.4213 (5)	2.4288 (12)
Cu—P1	2.2602 (6)	2.2849 (12)
Cu—P2	2.2380 (5)	2.2594 (12)
C1—S1	1.714 (2)	1.709 (4)
C1—S2	1.717 (2)	1.702 (4)
S1—Cu—S2	75.264 (18)	74.76 (4)
S1—Cu—P1	110.96 (2)	109.85 (5)
S1—Cu—P2	109.81 (2)	112.35 (4)
S2—Cu—P1	103.74 (2)	102.50 (4)
S2—Cu—P2	123.17 (2)	122.04 (5)
P1—Cu—P2	123.65 (2)	124.52 (4)

Note: (*a*) Jian *et al.* (2000 ▸).

**Table 2:** Hydrogen-bond geometry (Å, °) *Cg*1 is the ring centroid of (C51–C56).

*D*—H⋯*A*	*D*—H	H⋯*A*	*D*⋯*A*	*D*—H⋯*A*
O2—H2*O*⋯O1	0.84	1.95	2.710 (3)	150
O1—H1*O*⋯O2^i^	0.86	1.97	2.697 (3)	142
C6—Cl3⋯*Cg*1	1.77 (1)	3.81 (1)	3.798 (3)	76 (1)

Symmetry code: (i) 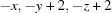.

**Table 3:** Experimental details

Crystal data	
Chemical formula	[Cu(C_5_H_5_NO_2_S_2_)(C_18_H_15_P)_2_]·CHCl_3_
*M*_r_	887.71
Crystal system, space group	Triclinic, *P* 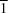
Temperature (K)	100
*a*, *b*, *c* (Å)	10.7271 (2), 13.5412 (2), 15.9361 (3)
α, β, γ (°)	67.747 (2), 87.126 (2), 72.826 (2)
*V* (Å^3^)	2041.92 (7)
*Z*	2
Radiation type	Mo *K*α
μ (mm^−1^)	0.95
Crystal size (mm)	0.44 × 0.24 × 0.19

Data collection	
Diffractometer	Rigaku SuperNova, Dual Mo at zero, AtlasS2
Absorption correction	Multi-scan (*CrysAlis PRO*; Rigaku Oxford Diffraction, 2015 ▸)
*T*_min_, *T*_max_	0.928, 1.000
No. of measured, independent and observed [*I* > 2σ(*I*)] reflections	78295, 11363, 10195
*R*_int_	0.029
(sin θ/λ)_max_ (Å^−1^)	0.708

Refinement	
*R*[*F* ^2^ > 2σ(*F* ^2^)], *wR*(*F* ^2^), *S*	0.045, 0.117, 1.03
No. of reflections	11363
No. of parameters	478
H-atom treatment	H-atom parameters constrained
Δρ_max_, Δρ_min_ (e Å^−3^)	1.97, −1.93

Computer programs: *CrysAlis PRO* (Rigaku Oxford Diffraction, 2015 ▸), *SHELXS* (Sheldrick, 2008 ▸), *SHELXL2014* (Sheldrick, 2015 ▸), *ORTEP-3 for Windows* (Farrugia, 2012 ▸), *QMol* (Gans & Shalloway, 2001 ▸), *DIAMOND* (Brandenburg, 2006 ▸) and *publCIF* (Westrip, 2010 ▸).

## supplementary crystallographic information

### Crystal data

**Table:**

[Cu(C_5_H_5_NO_2_S_2_)(C_18_H_15_P)_2_]·CHCl_3_	*Z* = 2
*M**_r_* = 887.71	*F*(000) = 916
Triclinic, *P*1	*D*_x_ = 1.444 Mg m^−^^3^
*a* = 10.7271 (2) Å	Mo *K*α radiation, λ = 0.71073 Å
*b* = 13.5412 (2) Å	Cell parameters from 38605 reflections
*c* = 15.9361 (3) Å	θ = 3.1–30.1°
α = 67.747 (2)°	µ = 0.95 mm^−^^1^
β = 87.126 (2)°	*T* = 100 K
γ = 72.826 (2)°	Prism, colourless
*V* = 2041.92 (7) Å^3^	0.44 × 0.24 × 0.19 mm

### Data collection

**Table:**

Rigaku SuperNova, Dual Mo at zero, AtlasS2 diffractometer	11363 independent reflections
Radiation source: micro-focus sealed X-ray tube, SuperNova (Mo) X-ray Source	10195 reflections with *I* > 2σ(*I*)
Mirror monochromator	*R*_int_ = 0.029
Detector resolution: 5.2303 pixels mm^-1^	θ_max_ = 30.2°, θ_min_ = 2.4°
ω scans	*h* = −14→14
Absorption correction: multi-scan (CrysAlis PRO; Rigaku Oxford Diffraction, 2015)	*k* = −19→18
*T*_min_ = 0.928, *T*_max_ = 1.000	*l* = −22→21
78295 measured reflections	

### Refinement

**Table:**

Refinement on *F*^2^	0 restraints
Least-squares matrix: full	Hydrogen site location: mixed
*R*[*F*^2^ > 2σ(*F*^2^)] = 0.045	H-atom parameters constrained
*wR*(*F*^2^) = 0.117	*w* = 1/[σ^2^(*F*_o_^2^) + (0.0542*P*)^2^ + 3.7972*P*] where *P* = (*F*_o_^2^ + 2*F*_c_^2^)/3
*S* = 1.03	(Δ/σ)_max_ = 0.002
11363 reflections	Δρ_max_ = 1.97 e Å^−^^3^
478 parameters	Δρ_min_ = −1.93 e Å^−^^3^

### Special details

**Table:**

Geometry. All esds (except the esd in the dihedral angle between two l.s. planes) are estimated using the full covariance matrix. The cell esds are taken into account individually in the estimation of esds in distances, angles and torsion angles; correlations between esds in cell parameters are only used when they are defined by crystal symmetry. An approximate (isotropic) treatment of cell esds is used for estimating esds involving l.s. planes.

### Fractional atomic coordinates and isotropic or equivalent isotropic displacement parameters (Å^2^)

**Table:**

	*x*	*y*	*z*	*U*_iso_*/*U*_eq_	
Cu	0.36192 (2)	0.77121 (2)	0.71076 (2)	0.01300 (7)	
S1	0.19989 (5)	0.72501 (4)	0.81318 (3)	0.01687 (10)	
S2	0.18227 (5)	0.94266 (4)	0.66887 (3)	0.01434 (10)	
P1	0.51698 (5)	0.80568 (4)	0.77705 (3)	0.01311 (10)	
P2	0.40667 (5)	0.66266 (4)	0.62988 (3)	0.01279 (10)	
O1	−0.0657 (2)	1.09492 (18)	0.86819 (14)	0.0388 (5)	
H1O	−0.0221	1.1248	0.8899	0.058*	
O2	−0.0552 (2)	0.89051 (18)	0.99446 (14)	0.0379 (5)	
H2O	−0.0317	0.9473	0.9646	0.057*	
N1	0.00449 (17)	0.90924 (16)	0.79337 (12)	0.0179 (3)	
C1	0.11675 (19)	0.86434 (16)	0.76152 (13)	0.0138 (3)	
C2	−0.0662 (2)	1.02862 (19)	0.74859 (15)	0.0222 (4)	
H2A	−0.1600	1.0404	0.7600	0.027*	
H2B	−0.0588	1.0517	0.6822	0.027*	
C3	−0.0154 (3)	1.1018 (2)	0.78121 (17)	0.0274 (5)	
H3A	0.0814	1.0761	0.7870	0.033*	
H3B	−0.0444	1.1802	0.7370	0.033*	
C4	−0.0538 (2)	0.8356 (2)	0.86728 (15)	0.0244 (5)	
H4A	−0.0415	0.7655	0.8574	0.029*	
H4B	−0.1491	0.8725	0.8635	0.029*	
C5	0.0018 (2)	0.8061 (2)	0.96199 (16)	0.0276 (5)	
H5A	−0.0136	0.7356	1.0035	0.033*	
H5B	0.0974	0.7937	0.9613	0.033*	
C11	0.4522 (2)	0.90455 (17)	0.83239 (13)	0.0155 (4)	
C12	0.3587 (2)	0.88068 (19)	0.89530 (15)	0.0193 (4)	
H12	0.3316	0.8164	0.9064	0.023*	
C13	0.3054 (2)	0.9506 (2)	0.94159 (15)	0.0223 (4)	
H13	0.2421	0.9338	0.9842	0.027*	
C14	0.3446 (2)	1.0450 (2)	0.92569 (16)	0.0239 (4)	
H14	0.3080	1.0928	0.9572	0.029*	
C15	0.4374 (3)	1.0687 (2)	0.86363 (16)	0.0264 (5)	
H15	0.4645	1.1330	0.8528	0.032*	
C16	0.4912 (2)	0.99887 (18)	0.81703 (15)	0.0214 (4)	
H16	0.5547	1.0158	0.7746	0.026*	
C21	0.6312 (2)	0.86383 (16)	0.69950 (13)	0.0140 (3)	
C22	0.5816 (2)	0.96097 (17)	0.62185 (14)	0.0186 (4)	
H22	0.4900	0.9973	0.6120	0.022*	
C23	0.6653 (2)	1.00436 (18)	0.55932 (15)	0.0221 (4)	
H23	0.6308	1.0706	0.5072	0.027*	
C24	0.7997 (2)	0.95133 (19)	0.57246 (15)	0.0212 (4)	
H24	0.8568	0.9818	0.5298	0.025*	
C25	0.8497 (2)	0.85403 (19)	0.64806 (14)	0.0196 (4)	
H25	0.9411	0.8171	0.6568	0.024*	
C26	0.7660 (2)	0.81042 (18)	0.71115 (14)	0.0169 (4)	
H26	0.8009	0.7436	0.7627	0.020*	
C31	0.6275 (2)	0.68849 (17)	0.86749 (13)	0.0162 (4)	
C32	0.6884 (2)	0.70275 (19)	0.93539 (15)	0.0218 (4)	
H32	0.6666	0.7743	0.9389	0.026*	
C33	0.7811 (2)	0.6126 (2)	0.99804 (16)	0.0268 (5)	
H33	0.8220	0.6228	1.0444	0.032*	
C34	0.8138 (3)	0.5084 (2)	0.99318 (17)	0.0300 (5)	
H34	0.8781	0.4474	1.0356	0.036*	
C35	0.7531 (3)	0.4929 (2)	0.9266 (2)	0.0347 (6)	
H35	0.7752	0.4211	0.9236	0.042*	
C36	0.6593 (2)	0.58267 (19)	0.86396 (18)	0.0267 (5)	
H36	0.6170	0.5716	0.8187	0.032*	
C41	0.48854 (19)	0.51623 (16)	0.69995 (14)	0.0156 (4)	
C42	0.5952 (2)	0.45035 (19)	0.67222 (16)	0.0220 (4)	
H42	0.6255	0.4805	0.6136	0.026*	
C43	0.6577 (2)	0.3406 (2)	0.73001 (18)	0.0272 (5)	
H43	0.7300	0.2961	0.7105	0.033*	
C44	0.6151 (2)	0.29608 (19)	0.81547 (18)	0.0273 (5)	
H44	0.6593	0.2219	0.8552	0.033*	
C45	0.5079 (3)	0.3600 (2)	0.84291 (17)	0.0273 (5)	
H45	0.4773	0.3288	0.9012	0.033*	
C46	0.4446 (2)	0.46945 (18)	0.78592 (16)	0.0223 (4)	
H46	0.3711	0.5127	0.8055	0.027*	
C51	0.26885 (19)	0.65508 (16)	0.57220 (13)	0.0135 (3)	
C52	0.2709 (2)	0.55962 (17)	0.55691 (14)	0.0169 (4)	
H52	0.3427	0.4937	0.5812	0.020*	
C53	0.1684 (2)	0.56083 (18)	0.50627 (15)	0.0196 (4)	
H53	0.1702	0.4958	0.4960	0.024*	
C54	0.0630 (2)	0.65717 (19)	0.47064 (15)	0.0207 (4)	
H54	−0.0067	0.6581	0.4355	0.025*	
C55	0.0595 (2)	0.75232 (18)	0.48645 (15)	0.0192 (4)	
H55	−0.0125	0.8181	0.4622	0.023*	
C56	0.16193 (19)	0.75072 (17)	0.53791 (14)	0.0158 (4)	
H56	0.1588	0.8151	0.5496	0.019*	
C61	0.51483 (19)	0.69669 (16)	0.53792 (14)	0.0150 (4)	
C62	0.4884 (2)	0.70661 (17)	0.44993 (14)	0.0179 (4)	
H62	0.4136	0.6900	0.4363	0.021*	
C63	0.5711 (2)	0.74078 (18)	0.38150 (16)	0.0225 (4)	
H63	0.5526	0.7468	0.3218	0.027*	
C64	0.6801 (2)	0.7659 (2)	0.40050 (18)	0.0266 (5)	
H64	0.7356	0.7901	0.3538	0.032*	
C65	0.7076 (2)	0.7553 (2)	0.4883 (2)	0.0295 (5)	
H65	0.7827	0.7716	0.5018	0.035*	
C66	0.6258 (2)	0.7211 (2)	0.55654 (17)	0.0237 (5)	
H66	0.6454	0.7141	0.6164	0.028*	
C6	0.0552 (3)	0.4509 (2)	0.7262 (2)	0.0354 (6)	
H6	0.0901	0.4682	0.6646	0.042*	
Cl1	0.14649 (11)	0.31507 (7)	0.79474 (7)	0.0593 (3)	
Cl2	−0.10822 (9)	0.46142 (12)	0.71310 (9)	0.0740 (4)	
Cl3	0.07466 (6)	0.55046 (5)	0.76719 (4)	0.03169 (13)	

### Atomic displacement parameters (Å^2^)

**Table:**

	*U*^11^	*U*^22^	*U*^33^	*U*^12^	*U*^13^	*U*^23^
Cu	0.01265 (12)	0.01232 (11)	0.01406 (12)	−0.00179 (8)	0.00184 (8)	−0.00664 (9)
S1	0.0200 (2)	0.0124 (2)	0.0169 (2)	−0.00543 (18)	0.00520 (18)	−0.00421 (17)
S2	0.0153 (2)	0.0118 (2)	0.0124 (2)	−0.00113 (16)	0.00266 (16)	−0.00311 (16)
P1	0.0136 (2)	0.0124 (2)	0.0123 (2)	−0.00270 (17)	0.00066 (17)	−0.00431 (17)
P2	0.0117 (2)	0.0117 (2)	0.0150 (2)	−0.00122 (17)	0.00111 (17)	−0.00678 (18)
O1	0.0411 (11)	0.0450 (12)	0.0303 (10)	0.0017 (9)	0.0000 (8)	−0.0252 (9)
O2	0.0432 (11)	0.0421 (11)	0.0300 (10)	−0.0052 (9)	0.0066 (8)	−0.0216 (9)
N1	0.0131 (8)	0.0227 (9)	0.0159 (8)	−0.0025 (7)	0.0033 (6)	−0.0078 (7)
C1	0.0134 (8)	0.0163 (9)	0.0125 (8)	−0.0043 (7)	0.0015 (7)	−0.0065 (7)
C2	0.0161 (9)	0.0255 (11)	0.0198 (10)	0.0043 (8)	−0.0011 (8)	−0.0107 (8)
C3	0.0300 (12)	0.0218 (11)	0.0257 (11)	0.0060 (9)	−0.0041 (9)	−0.0136 (9)
C4	0.0171 (10)	0.0363 (13)	0.0199 (10)	−0.0105 (9)	0.0076 (8)	−0.0097 (9)
C5	0.0244 (11)	0.0366 (13)	0.0182 (10)	−0.0062 (10)	0.0046 (8)	−0.0090 (9)
C11	0.0166 (9)	0.0161 (9)	0.0135 (9)	−0.0027 (7)	−0.0002 (7)	−0.0069 (7)
C12	0.0188 (10)	0.0219 (10)	0.0200 (10)	−0.0076 (8)	0.0039 (8)	−0.0103 (8)
C13	0.0220 (10)	0.0269 (11)	0.0194 (10)	−0.0055 (9)	0.0044 (8)	−0.0119 (9)
C14	0.0300 (12)	0.0234 (11)	0.0194 (10)	−0.0039 (9)	0.0027 (9)	−0.0126 (9)
C15	0.0400 (13)	0.0209 (10)	0.0233 (11)	−0.0130 (10)	0.0068 (10)	−0.0117 (9)
C16	0.0284 (11)	0.0194 (10)	0.0190 (10)	−0.0094 (8)	0.0055 (8)	−0.0090 (8)
C21	0.0178 (9)	0.0127 (8)	0.0120 (8)	−0.0049 (7)	0.0004 (7)	−0.0050 (7)
C22	0.0230 (10)	0.0134 (9)	0.0152 (9)	−0.0008 (7)	0.0012 (8)	−0.0046 (7)
C23	0.0335 (12)	0.0135 (9)	0.0153 (9)	−0.0054 (8)	0.0040 (8)	−0.0025 (7)
C24	0.0311 (11)	0.0200 (10)	0.0169 (10)	−0.0132 (9)	0.0084 (8)	−0.0083 (8)
C25	0.0191 (10)	0.0245 (10)	0.0171 (9)	−0.0087 (8)	0.0029 (8)	−0.0085 (8)
C26	0.0167 (9)	0.0187 (9)	0.0137 (9)	−0.0050 (7)	0.0002 (7)	−0.0047 (7)
C31	0.0153 (9)	0.0159 (9)	0.0132 (9)	−0.0052 (7)	0.0003 (7)	−0.0006 (7)
C32	0.0241 (11)	0.0226 (10)	0.0153 (9)	−0.0067 (8)	−0.0009 (8)	−0.0035 (8)
C33	0.0274 (12)	0.0311 (12)	0.0164 (10)	−0.0105 (10)	−0.0048 (8)	−0.0009 (9)
C34	0.0268 (12)	0.0241 (11)	0.0247 (12)	−0.0076 (9)	−0.0063 (9)	0.0073 (9)
C35	0.0373 (14)	0.0151 (10)	0.0410 (15)	−0.0023 (10)	−0.0130 (12)	−0.0012 (10)
C36	0.0292 (12)	0.0164 (10)	0.0299 (12)	−0.0050 (9)	−0.0087 (9)	−0.0038 (9)
C41	0.0146 (9)	0.0137 (8)	0.0183 (9)	−0.0020 (7)	−0.0018 (7)	−0.0072 (7)
C42	0.0199 (10)	0.0190 (10)	0.0232 (11)	0.0006 (8)	0.0000 (8)	−0.0085 (8)
C43	0.0213 (11)	0.0186 (10)	0.0339 (13)	0.0049 (8)	−0.0025 (9)	−0.0093 (9)
C44	0.0259 (11)	0.0158 (10)	0.0322 (12)	−0.0018 (8)	−0.0085 (9)	−0.0027 (9)
C45	0.0308 (12)	0.0188 (10)	0.0263 (12)	−0.0079 (9)	0.0009 (9)	−0.0017 (9)
C46	0.0218 (10)	0.0170 (10)	0.0249 (11)	−0.0042 (8)	0.0041 (8)	−0.0062 (8)
C51	0.0126 (8)	0.0146 (8)	0.0125 (8)	−0.0039 (7)	0.0032 (6)	−0.0049 (7)
C52	0.0156 (9)	0.0154 (9)	0.0198 (9)	−0.0023 (7)	0.0013 (7)	−0.0083 (7)
C53	0.0198 (10)	0.0201 (10)	0.0227 (10)	−0.0072 (8)	0.0031 (8)	−0.0116 (8)
C54	0.0156 (9)	0.0260 (11)	0.0214 (10)	−0.0068 (8)	0.0002 (8)	−0.0096 (8)
C55	0.0138 (9)	0.0177 (9)	0.0219 (10)	−0.0013 (7)	−0.0002 (7)	−0.0054 (8)
C56	0.0143 (9)	0.0137 (9)	0.0186 (9)	−0.0030 (7)	0.0035 (7)	−0.0064 (7)
C61	0.0131 (8)	0.0117 (8)	0.0209 (9)	−0.0018 (7)	0.0043 (7)	−0.0087 (7)
C62	0.0174 (9)	0.0152 (9)	0.0195 (10)	−0.0038 (7)	0.0037 (7)	−0.0060 (7)
C63	0.0245 (11)	0.0178 (10)	0.0205 (10)	−0.0044 (8)	0.0066 (8)	−0.0043 (8)
C64	0.0239 (11)	0.0217 (11)	0.0373 (13)	−0.0098 (9)	0.0165 (10)	−0.0142 (10)
C65	0.0198 (11)	0.0349 (13)	0.0484 (15)	−0.0146 (10)	0.0147 (10)	−0.0284 (12)
C66	0.0177 (10)	0.0309 (12)	0.0332 (12)	−0.0088 (9)	0.0075 (9)	−0.0231 (10)
C6	0.0351 (14)	0.0380 (14)	0.0356 (14)	−0.0135 (12)	−0.0031 (11)	−0.0143 (12)
Cl1	0.0739 (6)	0.0376 (4)	0.0688 (6)	−0.0043 (4)	−0.0159 (5)	−0.0297 (4)
Cl2	0.0320 (4)	0.1170 (9)	0.1089 (9)	−0.0168 (5)	−0.0041 (5)	−0.0847 (8)
Cl3	0.0340 (3)	0.0258 (3)	0.0323 (3)	−0.0095 (2)	−0.0041 (2)	−0.0067 (2)

### Geometric parameters (Å, º)

**Table:**

Cu—P2	2.2380 (5)	C26—H26	0.9500
Cu—P1	2.2602 (6)	C31—C32	1.392 (3)
Cu—S1	2.3791 (6)	C31—C36	1.394 (3)
Cu—S2	2.4213 (5)	C32—C33	1.391 (3)
S1—C1	1.714 (2)	C32—H32	0.9500
S2—C1	1.717 (2)	C33—C34	1.381 (4)
P1—C31	1.824 (2)	C33—H33	0.9500
P1—C21	1.825 (2)	C34—C35	1.383 (4)
P1—C11	1.827 (2)	C34—H34	0.9500
P2—C51	1.827 (2)	C35—C36	1.395 (3)
P2—C61	1.828 (2)	C35—H35	0.9500
P2—C41	1.828 (2)	C36—H36	0.9500
O1—C3	1.442 (3)	C41—C42	1.395 (3)
O1—H1O	0.8576	C41—C46	1.398 (3)
O2—C5	1.397 (3)	C42—C43	1.394 (3)
O2—H2O	0.8400	C42—H42	0.9500
N1—C1	1.348 (3)	C43—C44	1.380 (4)
N1—C2	1.468 (3)	C43—H43	0.9500
N1—C4	1.476 (3)	C44—C45	1.382 (4)
C2—C3	1.513 (3)	C44—H44	0.9500
C2—H2A	0.9900	C45—C46	1.390 (3)
C2—H2B	0.9900	C45—H45	0.9500
C3—H3A	0.9900	C46—H46	0.9500
C3—H3B	0.9900	C51—C56	1.393 (3)
C4—C5	1.512 (3)	C51—C52	1.397 (3)
C4—H4A	0.9900	C52—C53	1.389 (3)
C4—H4B	0.9900	C52—H52	0.9500
C5—H5A	0.9900	C53—C54	1.391 (3)
C5—H5B	0.9900	C53—H53	0.9500
C11—C16	1.391 (3)	C54—C55	1.393 (3)
C11—C12	1.400 (3)	C54—H54	0.9500
C12—C13	1.391 (3)	C55—C56	1.394 (3)
C12—H12	0.9500	C55—H55	0.9500
C13—C14	1.391 (3)	C56—H56	0.9500
C13—H13	0.9500	C61—C62	1.393 (3)
C14—C15	1.386 (3)	C61—C66	1.398 (3)
C14—H14	0.9500	C62—C63	1.397 (3)
C15—C16	1.395 (3)	C62—H62	0.9500
C15—H15	0.9500	C63—C64	1.387 (3)
C16—H16	0.9500	C63—H63	0.9500
C21—C26	1.399 (3)	C64—C65	1.390 (4)
C21—C22	1.402 (3)	C64—H64	0.9500
C22—C23	1.386 (3)	C65—C66	1.389 (3)
C22—H22	0.9500	C65—H65	0.9500
C23—C24	1.394 (3)	C66—H66	0.9500
C23—H23	0.9500	C6—Cl2	1.733 (3)
C24—C25	1.386 (3)	C6—Cl1	1.748 (3)
C24—H24	0.9500	C6—Cl3	1.771 (3)
C25—C26	1.392 (3)	C6—H6	1.0000
C25—H25	0.9500		
			
P2—Cu—P1	123.65 (2)	C24—C25—H25	120.0
P2—Cu—S1	109.81 (2)	C26—C25—H25	120.0
P1—Cu—S1	110.96 (2)	C25—C26—C21	120.80 (19)
P2—Cu—S2	123.17 (2)	C25—C26—H26	119.6
P1—Cu—S2	103.74 (2)	C21—C26—H26	119.6
S1—Cu—S2	75.264 (18)	C32—C31—C36	119.2 (2)
C1—S1—Cu	84.12 (7)	C32—C31—P1	122.25 (16)
C1—S2—Cu	82.75 (7)	C36—C31—P1	118.39 (16)
C31—P1—C21	101.95 (9)	C33—C32—C31	120.2 (2)
C31—P1—C11	101.78 (9)	C33—C32—H32	119.9
C21—P1—C11	104.44 (9)	C31—C32—H32	119.9
C31—P1—Cu	118.06 (7)	C34—C33—C32	120.3 (2)
C21—P1—Cu	114.57 (7)	C34—C33—H33	119.9
C11—P1—Cu	114.16 (7)	C32—C33—H33	119.9
C51—P2—C61	102.10 (9)	C33—C34—C35	120.0 (2)
C51—P2—C41	103.13 (9)	C33—C34—H34	120.0
C61—P2—C41	103.77 (9)	C35—C34—H34	120.0
C51—P2—Cu	117.50 (6)	C34—C35—C36	120.0 (2)
C61—P2—Cu	116.28 (7)	C34—C35—H35	120.0
C41—P2—Cu	112.27 (7)	C36—C35—H35	120.0
C3—O1—H1O	105.7	C31—C36—C35	120.2 (2)
C5—O2—H2O	109.4	C31—C36—H36	119.9
C1—N1—C2	120.12 (18)	C35—C36—H36	119.9
C1—N1—C4	119.48 (18)	C42—C41—C46	118.73 (19)
C2—N1—C4	120.09 (18)	C42—C41—P2	122.71 (17)
N1—C1—S1	120.50 (15)	C46—C41—P2	118.53 (16)
N1—C1—S2	122.12 (15)	C41—C42—C43	120.4 (2)
S1—C1—S2	117.38 (11)	C41—C42—H42	119.8
N1—C2—C3	113.01 (18)	C43—C42—H42	119.8
N1—C2—H2A	109.0	C44—C43—C42	120.3 (2)
C3—C2—H2A	109.0	C44—C43—H43	119.8
N1—C2—H2B	109.0	C42—C43—H43	119.8
C3—C2—H2B	109.0	C43—C44—C45	119.7 (2)
H2A—C2—H2B	107.8	C43—C44—H44	120.1
O1—C3—C2	108.3 (2)	C45—C44—H44	120.1
O1—C3—H3A	110.0	C44—C45—C46	120.5 (2)
C2—C3—H3A	110.0	C44—C45—H45	119.7
O1—C3—H3B	110.0	C46—C45—H45	119.7
C2—C3—H3B	110.0	C45—C46—C41	120.3 (2)
H3A—C3—H3B	108.4	C45—C46—H46	119.8
N1—C4—C5	114.9 (2)	C41—C46—H46	119.8
N1—C4—H4A	108.5	C56—C51—C52	119.51 (18)
C5—C4—H4A	108.5	C56—C51—P2	118.08 (15)
N1—C4—H4B	108.5	C52—C51—P2	122.29 (15)
C5—C4—H4B	108.5	C53—C52—C51	120.22 (19)
H4A—C4—H4B	107.5	C53—C52—H52	119.9
O2—C5—C4	111.6 (2)	C51—C52—H52	119.9
O2—C5—H5A	109.3	C52—C53—C54	120.1 (2)
C4—C5—H5A	109.3	C52—C53—H53	119.9
O2—C5—H5B	109.3	C54—C53—H53	119.9
C4—C5—H5B	109.3	C53—C54—C55	120.0 (2)
H5A—C5—H5B	108.0	C53—C54—H54	120.0
C16—C11—C12	119.11 (19)	C55—C54—H54	120.0
C16—C11—P1	124.41 (16)	C54—C55—C56	119.85 (19)
C12—C11—P1	116.47 (16)	C54—C55—H55	120.1
C13—C12—C11	120.4 (2)	C56—C55—H55	120.1
C13—C12—H12	119.8	C51—C56—C55	120.29 (19)
C11—C12—H12	119.8	C51—C56—H56	119.9
C14—C13—C12	120.2 (2)	C55—C56—H56	119.9
C14—C13—H13	119.9	C62—C61—C66	118.8 (2)
C12—C13—H13	119.9	C62—C61—P2	123.81 (16)
C15—C14—C13	119.6 (2)	C66—C61—P2	117.29 (16)
C15—C14—H14	120.2	C61—C62—C63	120.5 (2)
C13—C14—H14	120.2	C61—C62—H62	119.7
C14—C15—C16	120.4 (2)	C63—C62—H62	119.7
C14—C15—H15	119.8	C64—C63—C62	120.2 (2)
C16—C15—H15	119.8	C64—C63—H63	119.9
C11—C16—C15	120.3 (2)	C62—C63—H63	119.9
C11—C16—H16	119.9	C63—C64—C65	119.6 (2)
C15—C16—H16	119.9	C63—C64—H64	120.2
C26—C21—C22	118.63 (19)	C65—C64—H64	120.2
C26—C21—P1	122.28 (15)	C66—C65—C64	120.3 (2)
C22—C21—P1	118.91 (16)	C66—C65—H65	119.8
C23—C22—C21	120.4 (2)	C64—C65—H65	119.8
C23—C22—H22	119.8	C65—C66—C61	120.6 (2)
C21—C22—H22	119.8	C65—C66—H66	119.7
C22—C23—C24	120.4 (2)	C61—C66—H66	119.7
C22—C23—H23	119.8	Cl2—C6—Cl1	111.63 (17)
C24—C23—H23	119.8	Cl2—C6—Cl3	111.63 (17)
C25—C24—C23	119.7 (2)	Cl1—C6—Cl3	111.60 (15)
C25—C24—H24	120.1	Cl2—C6—H6	107.2
C23—C24—H24	120.1	Cl1—C6—H6	107.2
C24—C25—C26	120.0 (2)	Cl3—C6—H6	107.2
			
C2—N1—C1—S1	−179.88 (15)	P1—C31—C32—C33	−174.02 (18)
C4—N1—C1—S1	−6.2 (3)	C31—C32—C33—C34	0.3 (4)
C2—N1—C1—S2	0.1 (3)	C32—C33—C34—C35	−0.9 (4)
C4—N1—C1—S2	173.79 (16)	C33—C34—C35—C36	0.4 (4)
Cu—S1—C1—N1	−173.40 (17)	C32—C31—C36—C35	−1.3 (4)
Cu—S1—C1—S2	6.57 (10)	P1—C31—C36—C35	173.7 (2)
Cu—S2—C1—N1	173.49 (17)	C34—C35—C36—C31	0.7 (4)
Cu—S2—C1—S1	−6.48 (10)	C51—P2—C41—C42	96.01 (19)
C1—N1—C2—C3	−84.6 (2)	C61—P2—C41—C42	−10.2 (2)
C4—N1—C2—C3	101.8 (2)	Cu—P2—C41—C42	−136.55 (17)
N1—C2—C3—O1	−77.9 (2)	C51—P2—C41—C46	−85.86 (18)
C1—N1—C4—C5	83.8 (3)	C61—P2—C41—C46	167.95 (17)
C2—N1—C4—C5	−102.5 (2)	Cu—P2—C41—C46	41.58 (19)
N1—C4—C5—O2	82.1 (3)	C46—C41—C42—C43	−1.0 (3)
C31—P1—C11—C16	103.55 (19)	P2—C41—C42—C43	177.13 (18)
C21—P1—C11—C16	−2.2 (2)	C41—C42—C43—C44	−0.3 (4)
Cu—P1—C11—C16	−128.12 (17)	C42—C43—C44—C45	1.6 (4)
C31—P1—C11—C12	−75.31 (18)	C43—C44—C45—C46	−1.4 (4)
C21—P1—C11—C12	178.89 (16)	C44—C45—C46—C41	0.1 (4)
Cu—P1—C11—C12	53.01 (17)	C42—C41—C46—C45	1.1 (3)
C16—C11—C12—C13	0.2 (3)	P2—C41—C46—C45	−177.09 (19)
P1—C11—C12—C13	179.10 (17)	C61—P2—C51—C56	−94.07 (16)
C11—C12—C13—C14	0.0 (3)	C41—P2—C51—C56	158.48 (16)
C12—C13—C14—C15	−0.2 (4)	Cu—P2—C51—C56	34.40 (18)
C13—C14—C15—C16	0.2 (4)	C61—P2—C51—C52	82.03 (18)
C12—C11—C16—C15	−0.2 (3)	C41—P2—C51—C52	−25.42 (19)
P1—C11—C16—C15	−179.03 (18)	Cu—P2—C51—C52	−149.50 (15)
C14—C15—C16—C11	0.0 (4)	C56—C51—C52—C53	1.3 (3)
C31—P1—C21—C26	8.01 (19)	P2—C51—C52—C53	−174.71 (16)
C11—P1—C21—C26	113.67 (18)	C51—C52—C53—C54	−0.1 (3)
Cu—P1—C21—C26	−120.71 (16)	C52—C53—C54—C55	−0.6 (3)
C31—P1—C21—C22	−176.85 (16)	C53—C54—C55—C56	0.1 (3)
C11—P1—C21—C22	−71.19 (18)	C52—C51—C56—C55	−1.9 (3)
Cu—P1—C21—C22	54.43 (17)	P2—C51—C56—C55	174.34 (16)
C26—C21—C22—C23	−1.6 (3)	C54—C55—C56—C51	1.2 (3)
P1—C21—C22—C23	−176.94 (17)	C51—P2—C61—C62	−2.56 (19)
C21—C22—C23—C24	0.6 (3)	C41—P2—C61—C62	104.40 (18)
C22—C23—C24—C25	0.6 (3)	Cu—P2—C61—C62	−131.80 (16)
C23—C24—C25—C26	−0.9 (3)	C51—P2—C61—C66	173.12 (17)
C24—C25—C26—C21	−0.2 (3)	C41—P2—C61—C66	−79.91 (18)
C22—C21—C26—C25	1.4 (3)	Cu—P2—C61—C66	43.89 (18)
P1—C21—C26—C25	176.57 (16)	C66—C61—C62—C63	−0.3 (3)
C21—P1—C31—C32	79.67 (19)	P2—C61—C62—C63	175.35 (16)
C11—P1—C31—C32	−28.1 (2)	C61—C62—C63—C64	−0.4 (3)
Cu—P1—C31—C32	−153.85 (16)	C62—C63—C64—C65	0.9 (3)
C21—P1—C31—C36	−95.22 (19)	C63—C64—C65—C66	−0.7 (4)
C11—P1—C31—C36	157.05 (19)	C64—C65—C66—C61	0.0 (4)
Cu—P1—C31—C36	31.3 (2)	C62—C61—C66—C65	0.5 (3)
C36—C31—C32—C33	0.8 (3)	P2—C61—C66—C65	−175.44 (18)

### Hydrogen-bond geometry (Å, º)

*Cg*1 is the ring centroid of (C51–C56).

**Table:**

*D*—H···*A*	*D*—H	H···*A*	*D*···*A*	*D*—H···*A*
O2—H2*O*···O1	0.84	1.95	2.710 (3)	150
O1—H1*O*···O2^i^	0.86	1.97	2.697 (3)	142
C6—Cl3···*Cg*1	1.77 (1)	3.81 (1)	3.798 (3)	76 (1)

Symmetry code: (i) −*x*, −*y*+2, −*z*+2.
